# A Real Human Umbilical Cord Simulator Model for Emergency Umbilical Venous Catheter Placement Training

**DOI:** 10.7759/cureus.3544

**Published:** 2018-11-05

**Authors:** Taylor Sawyer, Megan Gray, Melinda Hendrickson, Elizabeth Jacobson, Rachel Umoren

**Affiliations:** 1 Pediatrics, University of Washington, Seattle, USA; 2 Neonatology, Seattle Childrens Hospital, Seattle, USA; 3 Pediatrics, Seattle Children's Hospital, Seattle , USA; 4 Pediatrics, Seattle Children's Hospital, Seattle, USA; 5 Pediatrics, University of Washington School of Medicine, Seattle, USA

**Keywords:** umbilicus and umbilical cord, umbilicus and umbilical cord, umbilical catheter, neonatal resuscitation, uvc, vascular access, emergency uvc, umbilical venous catheter, paediatric simulation, simulation

## Abstract

Emergency umbilical venous catheter placement is a critical procedure during newborn resuscitation. Providing training in this high-acuity and low-frequency procedure is important to optimize the skills of newborn resuscitation teams. Available simulators use simulated umbilical cords which are lower in fidelity than real human umbilical cords and may not provide optimal training. This technical report describes the creation and use of a real human umbilical cord simulator model for emergency umbilical venous catheter placement training. This low-cost model provides learners the opportunity to experience placing an emergency umbilical venous catheter in a real umbilical cord, providing a more realistic training model than currently available commercial simulators.

## Introduction

Emergency umbilical venous catheter (UVC) placement is the primary methods for obtaining intravenous access during newborn resuscitation. Placement of an emergency UVC provides a point for central intravenous access, which allows the resuscitation team to administer emergency medications and fluids. Both the Neonatal Resuscitation Program and the Newborn Life Support program prefer UVC placement over peripheral venous catheter insertion and intraosseous access [1, 2]. The UVC is preferred over a peripheral venous catheter because of the lower risk of extravasation of vasoconstrictive medications, like epinephrine, into the subcutaneous tissues. The UVC is preferred over intraosseous access due to limited data on the safety of intraosseous needle placement in neonates [3].

The need for emergency UVC placement during newborn resuscitation is rare. Emergency UVCs are only needed in approximately five in 10,000 newborn resuscitations [4-6]. Thus, emergency UVC is a high-acuity, low-frequency procedure for newborn resuscitation providers to master. Based on the limited opportunities to perform the procedure in clinical practice, and the critical importance of the procedure, newborn resuscitation educators rely on the use of simulation to teach emergency UVC placement.

Procedural skills training using simulation involves the use of task trainers or manikins that mimic human patients [7]. Using these task trainers and manikins, medical providers can practice rare procedures, like emergency UVC placement, to develop and maintain competency without harm to humans [8]. Emergency UVC training can use one of the two types of umbilical cord models: simulated umbilical cord and real umbilical cord. Prior research has shown that emergency UVC training with real umbilical cords has educational advantages for some learners [9]. In this Technical Report, we describe how to create a real human umbilical cord simulator model for emergency UVC placement training.

## Technical report

Equipment and supplies

- Fresh human umbilical cord (not frozen)

- Newborn simulator with a hole in the abdomen for the umbilical cord. Any brand of newborn simulator with a simulated umbilical cord will work.

- Baby bottle nipple and closure ring

- Rubber exam glove

- Umbilical clamp or Kelly clamp

Model assembly

**Figure 1:**
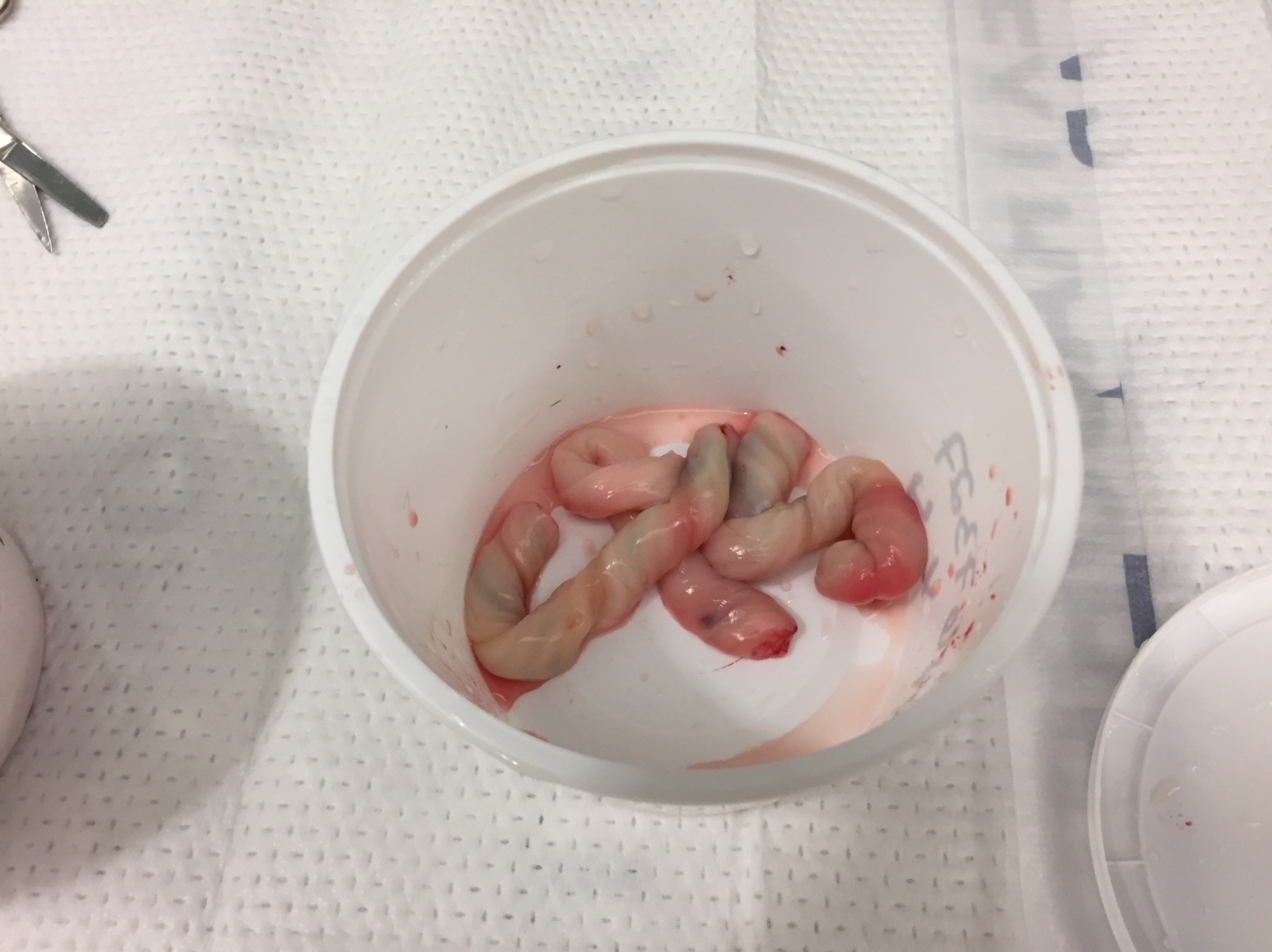
Human umbilical cords. Human umbilical cords in container.

Step 1. Clean the cords to remove any excess blood (Figure 1).

**Figure 2:**
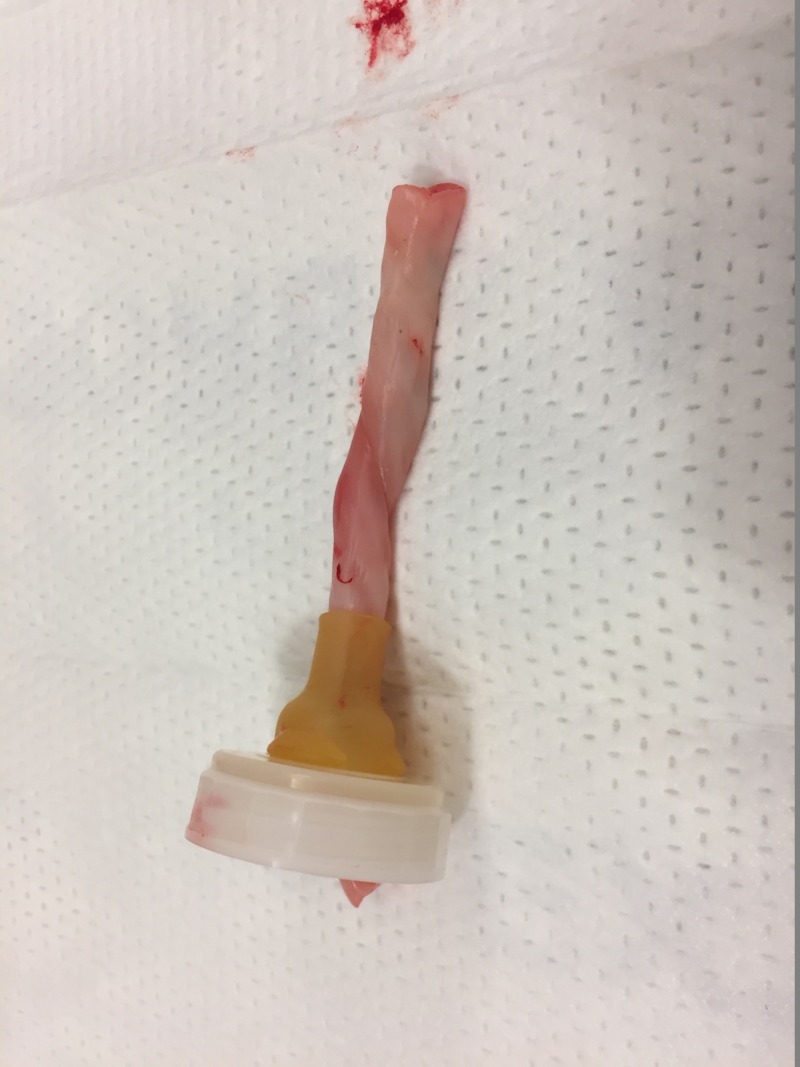
Umbilical cord in bottle nipple. Umbilical cord has been inserted into bottle nipple.

Step 2. Place the end of the umbilical cord inside a baby bottle nipple to create a base for the cord (Figure 2). This will require cutting the opening in the nipple to make it larger.

**Figure 3:**
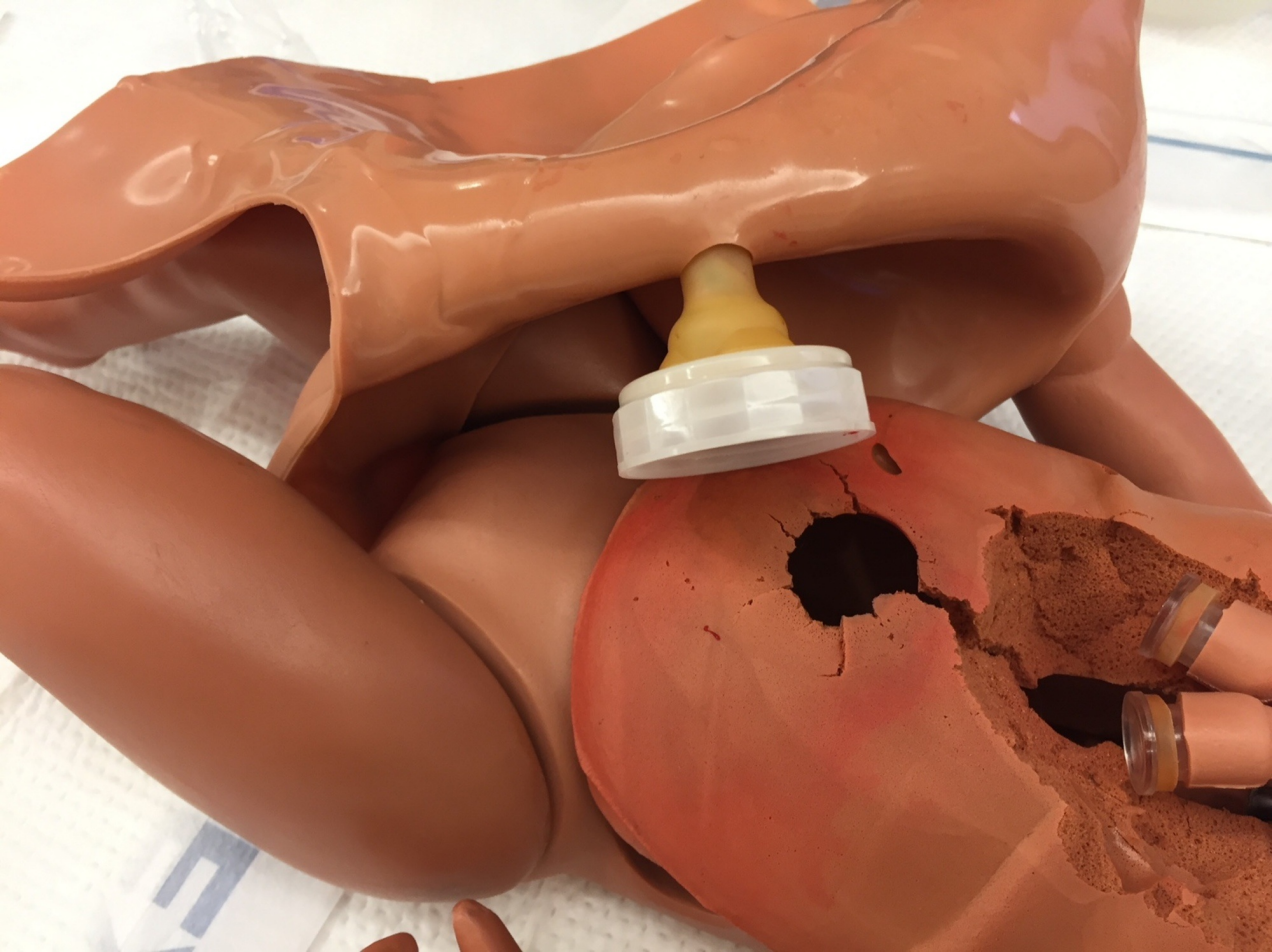
Umbilical cord inserted in simulator. Umbilical cord inserted into simulator with cord extending from hole in simulator's skin.

Step 3. Remove the outer skin from the manikin and place the umbilical cord through the hole (Figure 3).

**Figure 4:**
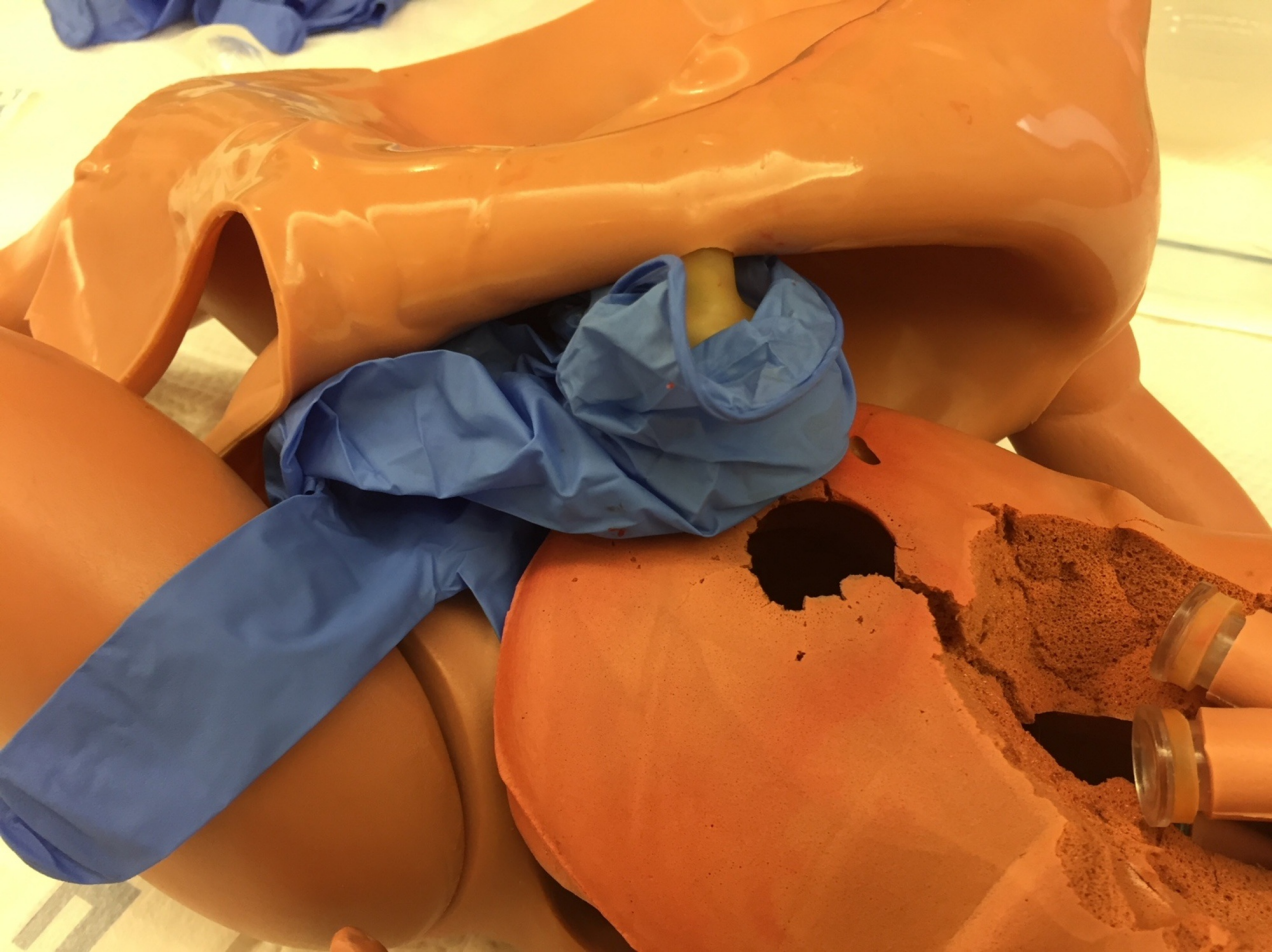
Glove around bottle nipple. Baby bottle nipple with the umbilical cord placed inside in a rubber glove to limit contamination.

Step 4. Place the baby bottle nipple with the umbilical cord inside in a rubber glove (Figure 4). This limits contamination of the inside of the manikin with blood and/or fluid.

**Figure 5:**
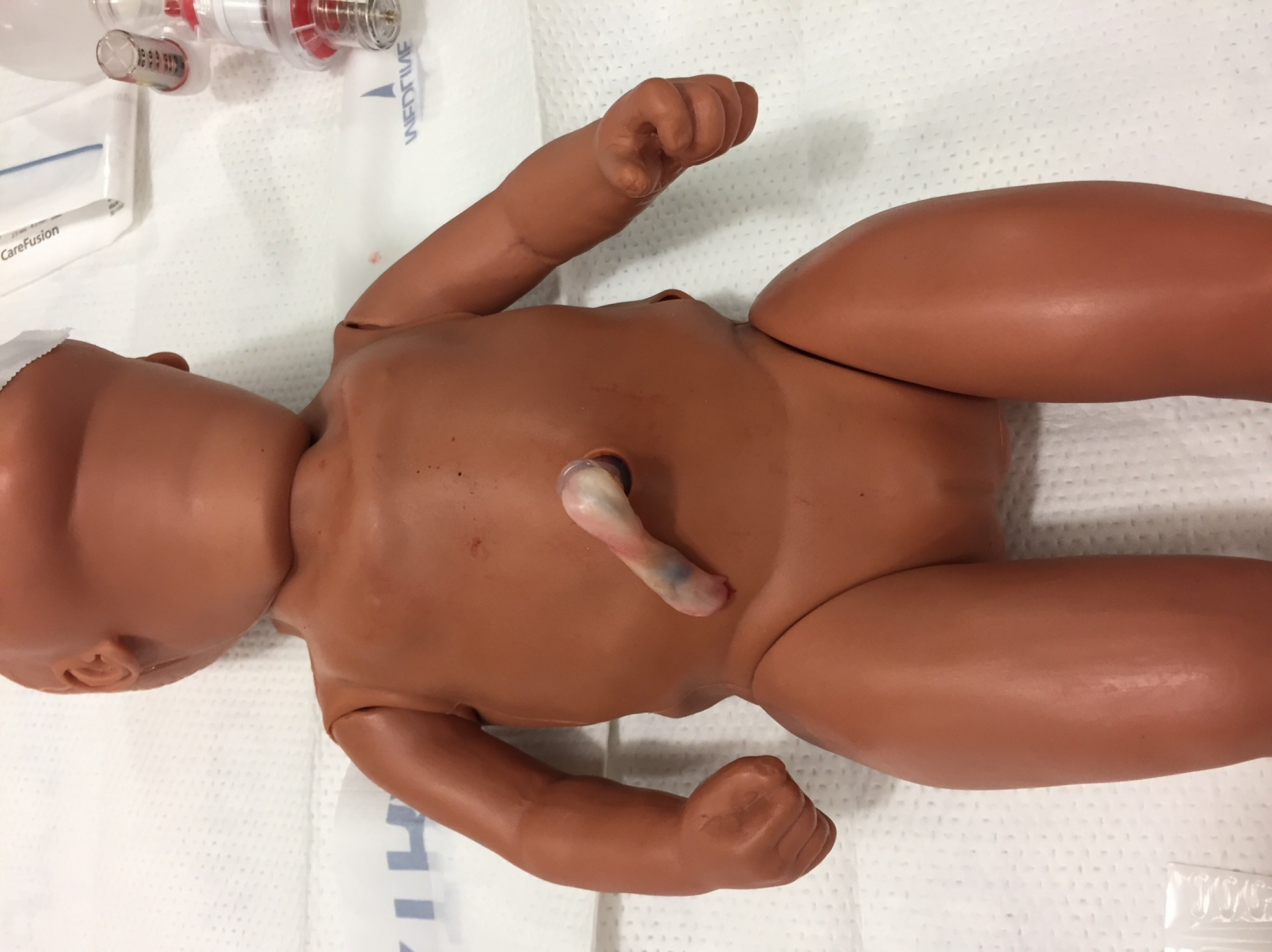
Umbilical cord integrated into manikin. Umbilical cord extends approximately 6 to 10 cm from the abdomen of the manikin.

Step 5. Replace the skin on the manikin and confirm the umbilical cord extends approximately 6 to 10 cm from the abdomen of the manikin (Figure 5).

**Figure 6:**
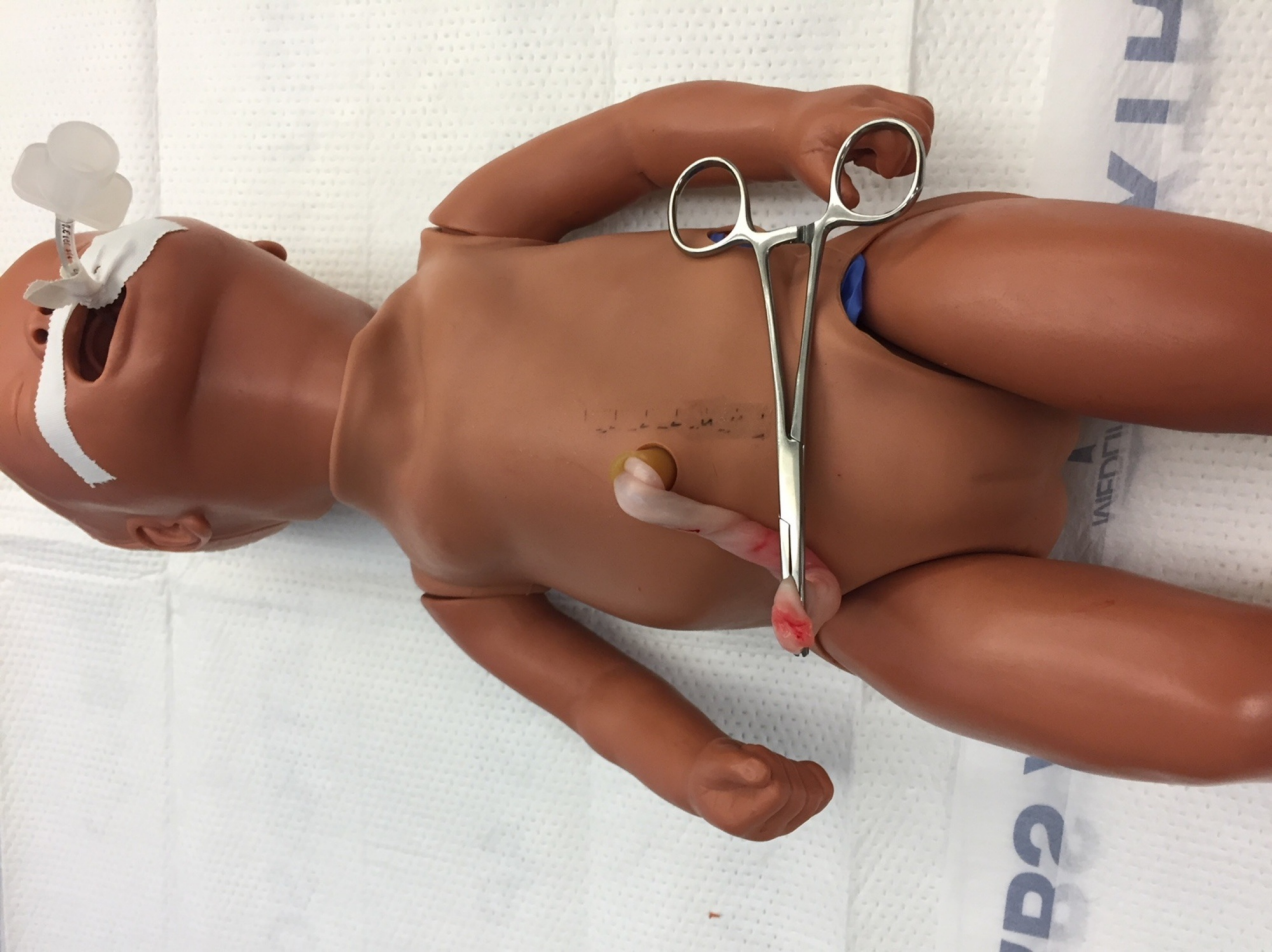
Clamp on cord. Clamp placed in the end of the umbilical cord to simulate a freshly clamped and cut umbilical cord.

Step 6. Place a clamp on the end of the umbilical cord to simulate a freshly clamped and cut umbilical cord (Figure 6).

**Figure 7:**
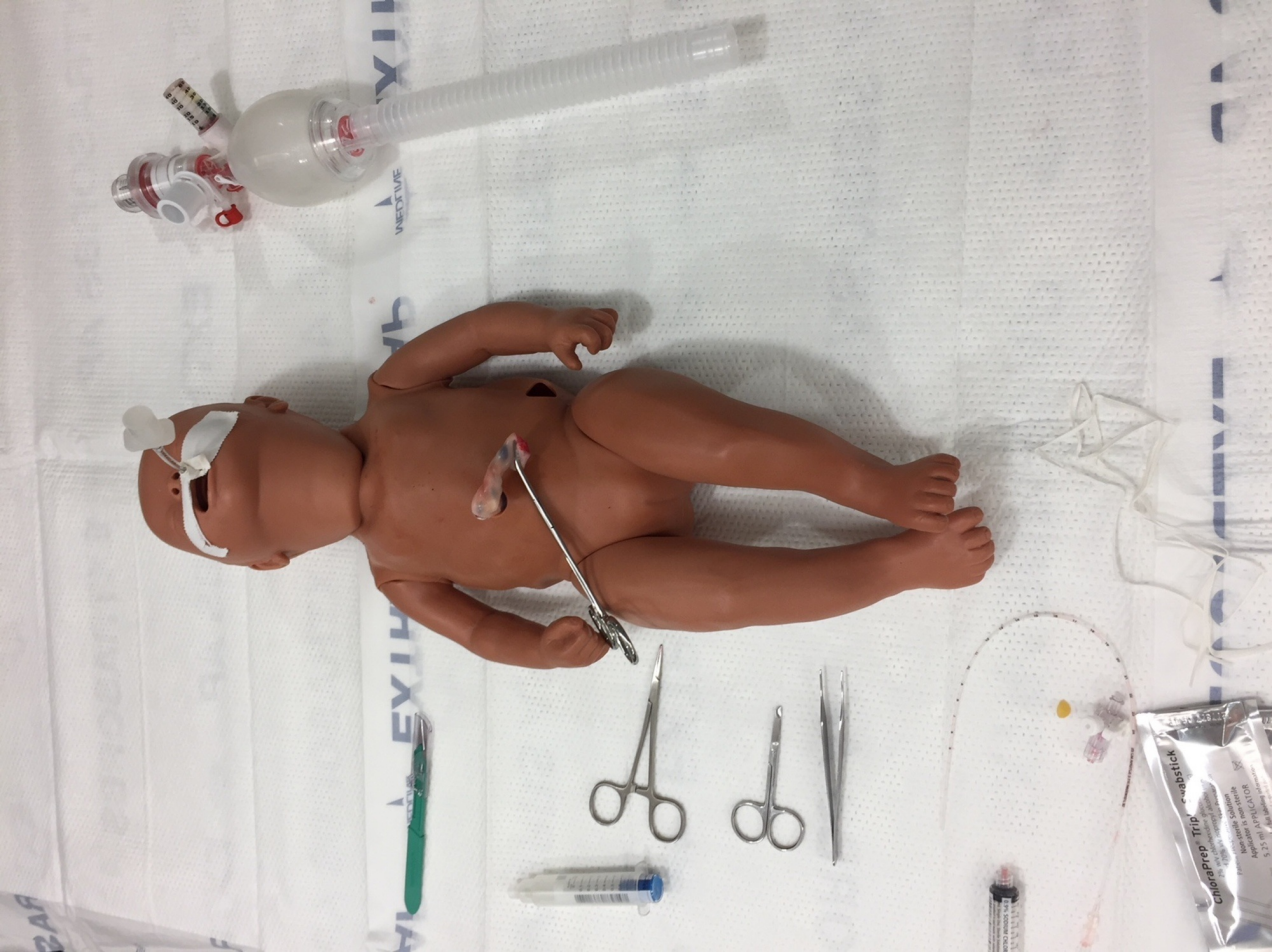
Real human umbilical cord simulator ready for use. Real human umbilical cord simulator model on a flat surface, with equipment needed for umbilical venous catheter (UVC) placement easily available.

Step 7. Place the real human umbilical cord simulator model on a flat surface, with equipment needed for UVC placement easily available (Figure 7).

Simulator use

**Figure 8:**
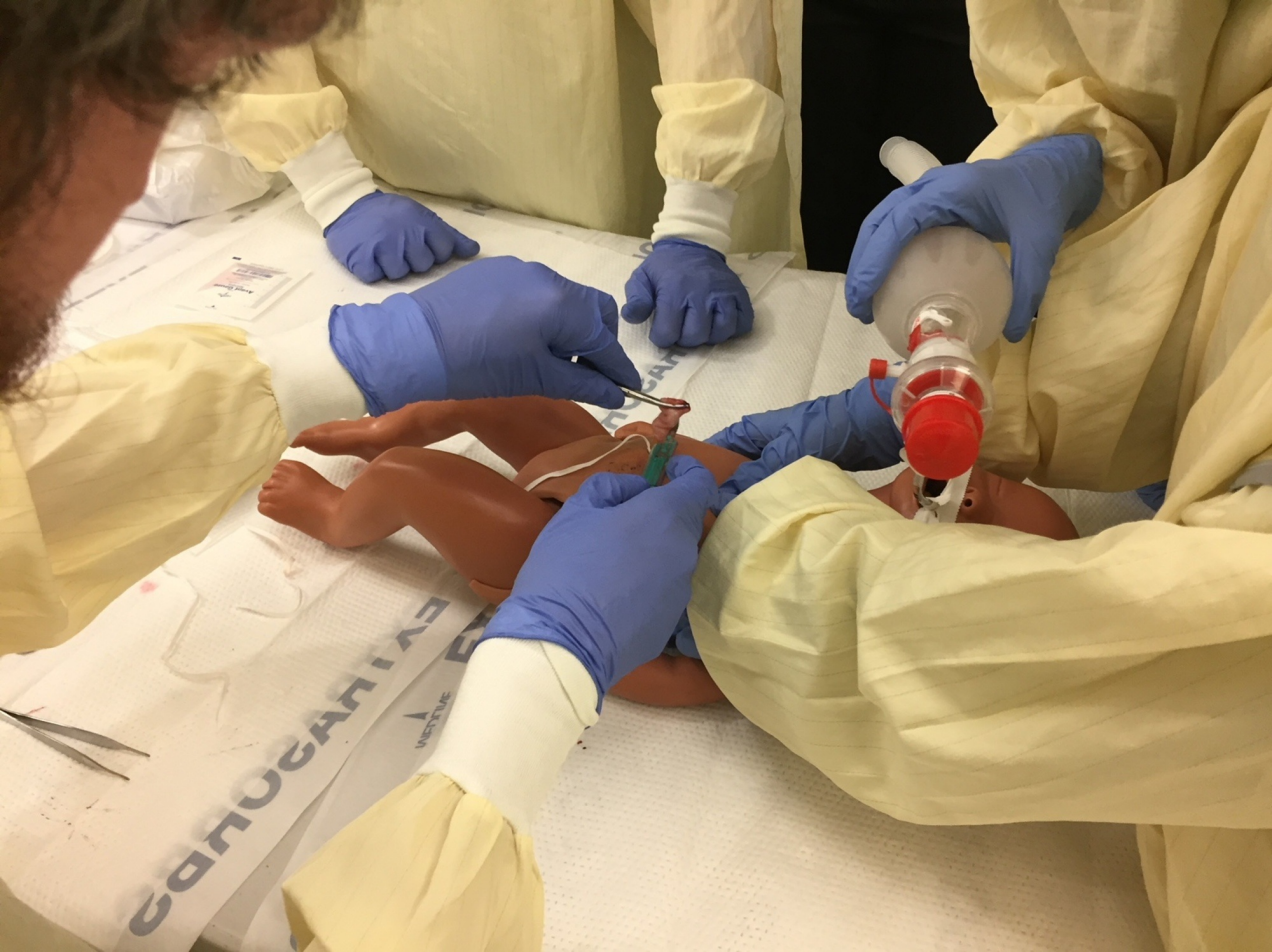
Team using real human umbilical cord simulator model. Team using real human umbilical cord simulator model in a training session.

When using this model, we have a team of three to four learners work together to simulate a newborn resuscitation in progress (Figure 8). We start the training session with the manikin already intubated. We ask the team to imagine that they are on a newborn resuscitation team actively resuscitating a full-term infant that was born with apnea and bradycardia that did not respond to positive pressure ventilation via face mask, endotracheal intubation, and chest compressions. Thus, the team has decided to place an emergency UVC to give intravenous epinephrine.

When the training starts, one team member provides ventilation through the endotracheal tube, one team member gives chest compressions, and one team member placed the emergency umbilical catheter. The other team member, if present, directs the other members and assists as needed. We ask that the team wear personal protection including gowns, gloves, face mask, and eye shields (Figure 8).

During the session the performance of the team is monitored by an educator who provides real-time feedback and coaching to the team. Once the first learner has placed the UVC, we have the team members rotate positions. We continue this rotation sequence until all team members have had the chance to place the emergency UVC. Once all team members have placed the catheter, we perform a team debriefing to discuss the team’s experiences placing the emergency UVC, address any observed performance issues, and answer any questions. Using this training methodology, we can cycle a team of four learners through the emergency UVC training station in approximately 30 minutes.

## Discussion

In this Technical Report, we described how to create a real human umbilical cord simulator model for emergency UVC placement training. The goal of the report is to provide educators interested in the use of a real umbilical cord training model easy to use instructions on how to create such a model for their learners. We believe there are benefits to the use of this real umbilical cord model for some learners.

Prior reports have examined different methods of emergency UVC training. Sawyer et al. used a cross-over study to compare the use of real versus simulated umbilical cords for emergency UVC placement in a group of pediatric residents [9]. They found that real umbilical cords were rated as having higher fidelity and were preferred over simulated umbilical cords by the study subjects. The time required to place an emergency UVC in a real cord was longer than in a simulated cord (153 ± 71 seconds in real umbilical cord model vs. 88 ± 35 seconds in simulated umbilical cord model; P < 0.001). The authors felt the difference was likely due to the increased time required to correctly identify and cannulate the umbilical vein in the real cord. There was no difference, however, in the time to place the UVC in the group that worked with simulated cords first. This suggests there are benefits to training with a simulated umbilical cord first, which allows the learner the ability to practice the individual steps of UVC placement and to refine their motor actions, before working with a real cord. Overall, these data suggest that training with both real and simulated umbilical cords has educational advantages for pediatric residents. Such a training paradigm conforms to recommendations to increase simulation fidelity as the level of training increases [10]. The model described here is the same model used in the investigation by Sawyer et al. [9].

There are several issues to consider when using real human umbilical cords for emergency UVC placement training. One issue is obtaining the umbilical cords. In our hospital, the cords are collected at the time of delivery by labor and delivery staff. They are then stored in the Pathology department. Our hospital has a standard procedure for checking out and returning the cords for educational use and there is no cost associated with the use. As umbilical cords are considered medical waste, no consent is needed for use. The collection, storage, and availability of umbilical cords for training may vary from hospital to hospital. Another issue is the potential of exposure to infectious disease inherent in working with human tissues. With the model described here, there can be blood leakage from the real umbilical cords onto the simulator and surrounding area. Cleaning the cords with water before placement into the model limits this issue but does not eliminate it entirely. Standard contact precautions must be followed when working with real umbilical cords. Personal protective equipment including masks, eye protection, gowns, and gloves should always be worn. Additionally, a system to link a specific segment of cord to the maternal medical record number should be in place. This allows analysis of infectious disease exposure risk in the event of a body fluid exposure or needle stick injury.

## Conclusions

In this Technical Report, we described how to create a real human umbilical cord simulator model for emergency UVC placement training. We believe the method we describe can be easily emulated by others. Based on prior studies, the use of real umbilical cords for emergency UVC training appears beneficial. Additional studies with translational clinical outcomes are needed to evaluate the effectiveness of emergency UVC placement training using the real umbilical cords model described here.
